# Non-isothermal crystallization, yellowing resistance and mechanical properties of heat-resistant nylon 10T/66/titania dioxide/glass fibre composites

**DOI:** 10.1039/c8ra10037c

**Published:** 2019-03-01

**Authors:** Bingxiao Liu, Guosheng Hu, Jingting Zhang, Wen Yan

**Affiliations:** Institute of Macromolecules and Bioengineering, School of Materials Science and Engineering, North University of China Taiyuan 030051 China; Public Service Platform for Science and Technology, Shenzhen Institutes of Advanced Technology, Chinese Academy of Sciences Shenzhen University Town, 1068 Xueyuan Avenue Shenzhen 518055 PR China

## Abstract

Herein, we report novel heat-resistant nylon 10T/66/titania dioxide/glass fibre (nylon 10T/66/TiO_2_/GF) composites based on as-synthesised nylon 10T/66, which is a copolymer of poly(decamethylene terephthalamide) (nylon 10T). The non-isothermal crystallization behaviors of nylon 10T/66 and nylon 10T/66/TiO_2_/GF composites were investigated by differential scanning calorimetry (DSC). Jeziorny and Mo equations were used to analyse the crystallization kinetics, whereas the Kissinger method was applied to calculate the activation energy. It turned out that the introduction of TiO_2_ and GF could accelerate the crystallization of nylon 10T/66 and exhibited an effective heterogeneous nucleation effect. In addition, we conducted yellowing resistance and mechanical property analysis of the nylon 10T/66/TiO_2_/GF composites. The above results successfully demonstrated that the heat-resistant nylon 10T/66/TiO_2_/GF composites possess higher crystallization temperature and crystallization rate, whiter color, and better yellowing resistance and mechanical properties than previously as-synthesised nylon 10T/66. Consequently, nylon 10T/66/TiO_2_/GF composites have great potential to be used as a heat-resistant engineering plastic.

## Abstract 2

Herein, we report novel heat-resistant nylon 10T/66/titania dioxide/glass fibre (nylon 10T/66/TiO_2_/GF) composites based on as-synthesised nylon 10T/66, which is a copolymer of poly(decamethylene terephthalamide) (nylon 10T).

## Graphical abstract

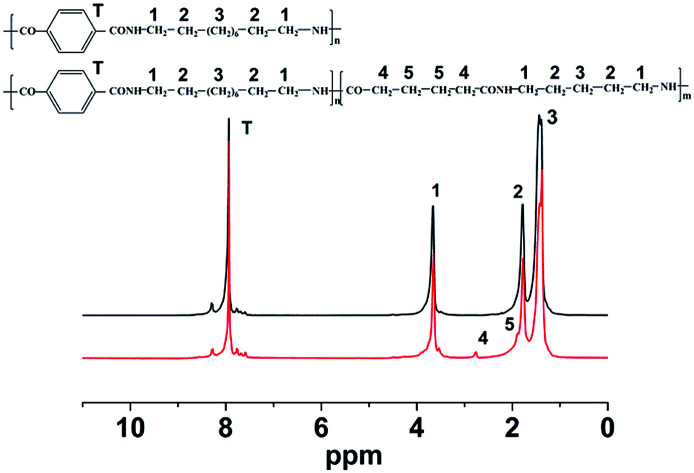

## 1. Introduction

Semi-aromatic nylon, combining the superior thermal stability of aromatic nylon and the excellent melt processability of aliphatic nylon, has been widely applied in the field of electronics (*e.g.*, connectors, surface mount devices and reflectors) and automobile parts (*e.g.*, powertrain components).^1,2^ Typical commercial semi-aromatic nylon includes poly(hexamethylene terephthalamide) (nylon 6T) copolymers (Dupont, Solvary *et al.*), poly(nonamethylene terephthalamide) (nylon 9T) (Kuraray) and nylon 10T (Kingfa and Zenong). Among them, nylon 10T is the only bio-based semi-aromatic heat-resistant nylon.^3^ However, the melting point of nylon 10T (316 °C) is relatively close to its initial decomposition temperature (350 °C) which makes conventional melt processing impractical.^4^ Consequently, we introduced the aliphatic nylon 66 chains into the backbones of nylon 10T, and obtained nylon 10T/66 copolymer,^5,6^ which possesses better melt processability.

However, the yellower color, poor yellowing resistance and mechanical properties limit the application of the neat nylon 10T/66. As we all know, blending is the most common and effective way to improve polymer performance.^7^

Glass fiber (GF), due to its high strength and low price, has become one of the most extensive reinforcement materials.^8,9^ Li *et al.* investigated the effect of GF addition on mechanical properties of poly(arylene ether nitriles), and found the tensile strength, flexural strength and izod impact strength of poly(arylene ether nitriles) were sharply increased in the presence of GF.^10^ Titania dioxide (TiO_2_) is applied as whiteners in a variety of polymeric composiions.^11^ Wang *et al.* added TiO_2_ into knitted fabric, which successfully improved its color and yellowing resistance.^12^

At present, many papers have studied the mechanical properties of GF reinforced nylon, and lots of researches have reported the improvement of the yellowing resistance of TiO_2_ to nylon, however, to the best of our knowledge, there has been no report that GF, TiO_2_ and nylon resin are ternary blended to simultaneously improve their mechanical properties and yellowing resistance properties. In this paper, we prepared nylon 10T/66/TiO_2_/GF by blending as-synthesised nylon 10T/66 with GF and TiO_2_. The morphological structures of nylon 10T/66/TiO_2_/GF were observed by scanning electron microscopy (SEM). In order to investigate the effect of GF and TiO_2_ on the crystallization of polymers and better understand the relationship between the structure and properties of materials, it is essential to study the crystallization kinetics, especially the non-isothermal crystallization kinetics, which is closer to the actual processing condition.^13,14^ The non-isothermal kinetics parameters of nylon 10T/66/TiO_2_/GF were carried out using the Jeziorny^15^ and the Mo equations.^16^ The non-isothermal crystallization activation energy was calculated by Kissinger method.^17^ In addition, the yellowing resistance and the mechanical properties of nylon 10T/66/TiO_2_/GF composites were also assessed and reported here.

## 2. Experimental

### 2.1 Materials

Decamethylenediamine (DA10), hexamethylene diamine (HMD) and adipic acid were supplied by Wuxi Yinda Nylons Co. Ltd. (Wuxi, China). The terephthalic acid (PTA) was purchased from Beijing Yanshan Lithification Chemical Co. Ltd. (Beijing, China). The benzoic acid (BA) was bought from Tianjin Kai Tong Chemical Reagent Co., Ltd (Tianjin, China). Taishan Fiberglass Inc. and DuPont provided the GF and TiO_2_ (Ti-pure R-103, rutile), respectively.

### 2.2 Synthesis of nylon 10T/66

DA10 (172.3 g, 1 mol), PTA (166.1 g, 1 mol), BA (4.6 g, 0.038 mol) and nylon 66 salt (13.1 g, 0.05 mol) were added into an autoclave. To reduce volatilization of diamine during polymerization, boiled distilled water (100 mL) was added. BA is used to control the molecular weight of copolymers. Then, the autoclave was purged with N_2_ for 5 min. At a pressure of about 0.4 MPa pressure and a 300 °C set point, the mixture temperature remained at 125 °C until all water evaporated. This stage promotes the homogenization of the reaction mixture, with almost no polymerization. When the reaction temperature reached 273 °C, meanwhile the pressure was up to 2.0 MPa, the heater set point was changed to 330 °C and the temperature was allowed to rise and hold at 280 °C. After allowing to react for 2 h, the pressure of the autoclave was gradually released to atmospheric pressure in 1 h and the reaction temperature of the sample was increased to 320 °C. Then the pressure of the autoclave was evacuated to −0.09 MPa. The reaction was continued for another 0.6 h and the copolymers were obtained.

### 2.3 Preparation of nylon 10T/66/TiO_2_, nylon 10T/66/GF and nylon 10T/66/TiO_2_/GF

Nylon 10T/66, TiO_2_ and GF were extruded from twin-screw extruder. Each region temperature was 300, 305, 305, and 295 °C, respectively. The screw speed was 40 rpm. The detailed dosages are presented in Table 1.

**Table 1:** Composition of nylon 10T/66/TiO_2_, nylon 10T/66/GF and nylon 10T/66/TiO_2_/GF

Samples	Nylon10T/66/g	TiO_2_/g	GF/g
Nylon 10T/66/TiO_2_	700	200	0
Nylon 10T/66/GF	700	200	0
Nylon 10T/66/TiO_2_/GF	700	200	200

### 2.4 Characterization of nylon 10T/66/TiO_2_/GF composites

^1^H NMR, Bruker DPX-400 at 400 MHz, was applied to identify the chemical structure of nylon 10T/66, using deuterated trifluoroacetic acid as solvents.

In order to improve conductivity, all samples were coated with gold before testing. The surface morphology was performed by cross-section scanning electron microscopy (SEM, MIRA3 FE-SEM, Czech) with an Oxford energy dispersive spectrometer (EDS).

DSC measurements were carried out on a Mettler 822e equipped with a STAR system, and calibrated with an indium standard. All measurements were under a nitrogen atmosphere (50 mL min^−1^). Sample with mass of 3 mg was heated from 25 °C to 320 °C at a rate of 50 °C min^−1^ and held this temperature for 5 min in order to eliminate the thermal history. After that, the sample was cooled to 30 °C at different cooling rates of 5, 10, 20 and 30 °C min^−1^, respectively. Record the non-isothermal crystallization curves as a function of time.

We injected the standard color plates using the injection moulding machine (HF-036). Then plates were put into the blast oven at the set temperature of 180 °C for 0 and 2 hours respectively. The CIELAB color parameters were performed under the Color i5 spectrophotometer. In the CIELAB system, the *L*, *b* denote the white-black value and yellow-blue of the materials, respectively.

The standard mechanical test samples of nylon 10T/66/TiO_2_, nylon 10T/66/GF and nylon 10T/66/TiO_2_/GF were prepared by an injection molding machine (HF-036). The tensile and bending properties were measured by Universal Testing Machine CMT6104 according to the ISO527 and ISO178. The impact property was tested on the basis of ISO179 by an impact testing machine XJU-22.

## 3. Results and discussion

### 3.1 Synthesis of nylon 10T/66

**Fig. 1:**
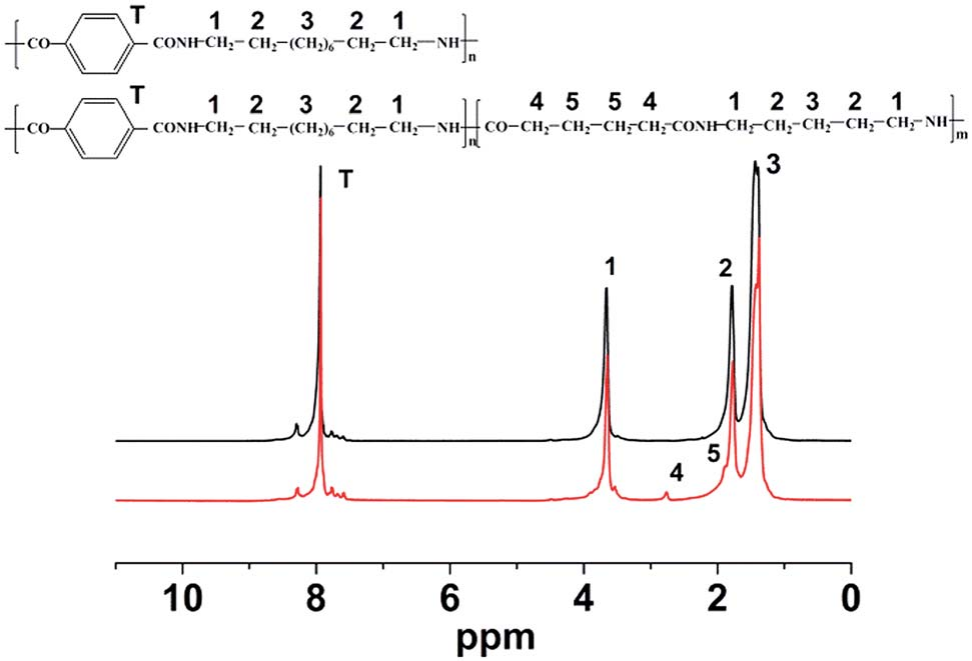
^1^H NMR spectra of nylon 10T/66.

The chemical structure of nylon 10T/66 is determined by ^1^H NMR spectra (Fig. 1). The chemical shifts, in the range of 3.71–3.62 ppm, originate from the protons of methylene adjacent to the NH group (position 1). The peaks at 1.82–1.75 ppm and 1.48–1.34 can be attributed to the position 2 and 3, respectively. The chemical shifts at 7.96–7.93 ppm correspond to the aromatic protons (position T). It is worth noting that the chemical shifts of nylon 10T/66 at 2.76 ppm and 1.89 ppm correspond to the positions 4 and 5, respectively, which indicates the formation of nylon 10T/66. After testing, the melting point of nylon 10T/66 (305 °C) is lower than that of neat nylon 10T (316 °C), suggesting that nylon 10T/66 possesses better processability.

### 3.2 SEM and EDS analysis of nylon 10T/66/TiO_2_/GF

**Fig. 2:**
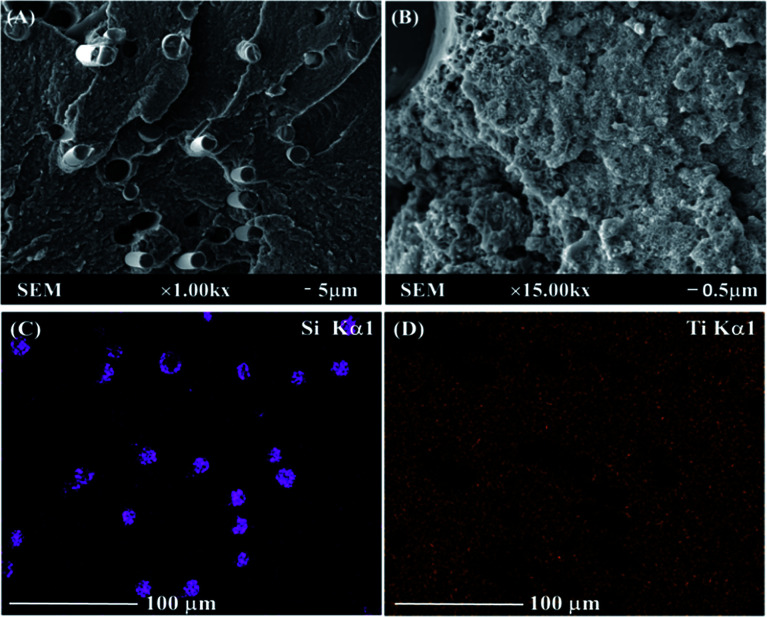
SEM and EDS photos of nylon 10T/66/TiO_2_/GF.

SEM and EDS images shown in Fig. 2 are applied to evaluate the dispersion of the GF and TiO_2_ in the nylon 10T/66 matrix. In Fig. 2A, the GF phases, which are cylindrical, are well wrapped by the nylon matrix. The white substances in Fig. 2B are TiO_2_, and it can be clearly seen that TiO_2_ is well dispersed in the matrix. SEM results indicated the excellent compatibility between the TiO_2_, GF and matrix resin in nylon 10T/66/TiO_2_/GF.

Fig. 2C and D represent the EDS dot map distribution images of the titanium and silicon in nylon 10T/66/TiO_2_/GF, respectively. As shown in these two figures, both titanium and silicon elements were uniformly distributed in the nylon matrix (silicon element is derived from GF), suggesting that the outstanding dispersion of the TiO_2_ and GF in nylon matrix resin, which is similar to the SEM results.

### 3.3 FT-IR analysis

**Fig. 3:**
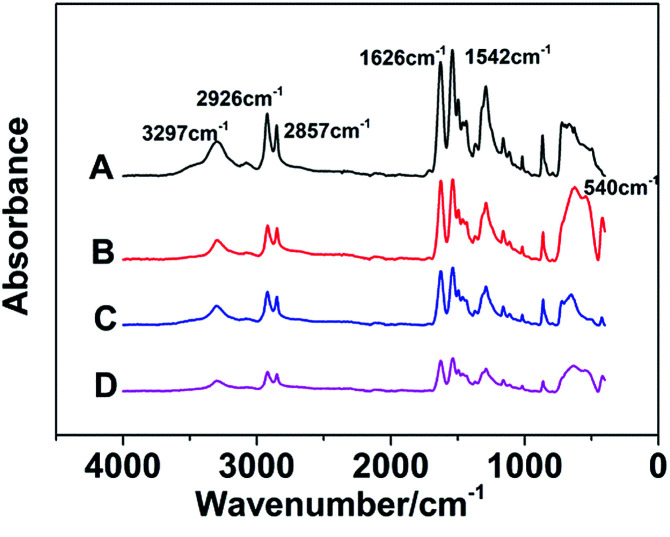
The FT-IR spectrums of nylon 10T/66 (A), nylon 10T/66/TiO_2_ (B), nylon 10T/66/GF (C) and nylon 10T/66/TiO_2_/GF (D).

The FT-IR spectrums of different samples are presented in Fig. 3. The absorbance peaks appeared at 3297 cm^−1^ are compounding to stretching vibration of N–H, and the peaks at 2926 cm^−1^ and 2857 cm^−1^ represent asymmetric and symmetrical stretching vibration of –CH_2_ respectively. Moreover, the absorption bands of amide can be reflected in these spectrums. In detail, the stretching vibration of C=O, C–N and C–CO are observed at 1626, 1380 and 1018 cm^−1^ respectively, and the in-plane flexural vibration of N–H is revealed at 1542 cm^−1^. The absorbance peaks appeared at 864 cm^−1^ and 624 cm^−1^ are compounding to out-of-plane flexural vibration of C–H. In addition, the absorbance peak at 540 cm^−1^ is caused by TiO_2_. These results of absorbance peaks prove that both TiO_2_ and GF are without influence upon the chemical bonding structure of nylon 10T/66.

### 3.4 Non-isothermal crystallization analysis

#### 3.4.1 Non-isothermal crystallization behaviors of samples

**Fig. 4:**
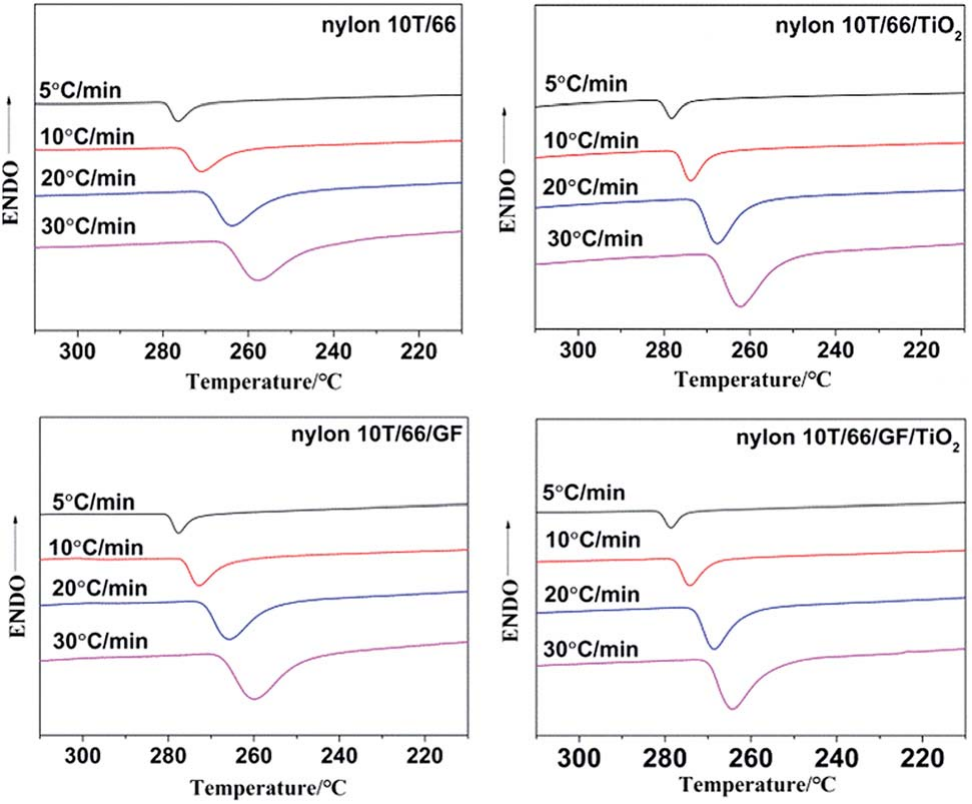
Non-isothermal crystallization plots of nylon 10T/66, nylon 10T/66/TiO_2_, nylon 10T/66/GF and nylon 10T/66/TiO_2_/GF.

The non-isothermal crystallization curves of nylon 10T/66, nylon 10T/66/TiO_2_, nylon 10T/66/GF and nylon 10T/66/TiO_2_/GF at various cooling rate are shown in Fig. 4, and the crystallization peak temperatures (*T*_p_) are summarized in Table 2. It could be clearly found from Fig. 4 that for all samples, the crystallization temperature decreases significantly and the temperature range becomes wider with increasing cooling rate, which is a common phenomenon of semi-crystalline polymers.^7,18^ This suggests that the molecular chain does not have enough time to overcome the barrier to form crystal nuclei and perfect crystals at higher cooling rate.^19,20^ According to the Table 2, for a fixed cooling rate, the crystallization temperature of neat nylon 10T/66 increases with the addition of TiO_2_ and GF, which indicates that TiO_2_ and GF play nucleating agent roles in accelerating the crystallization process of neat nylon 10T/66.^13^ Moreover, nylon 10T/66/TiO_2_/GF has the highest crystallization temperature, since TiO_2_ and GF have a synergistic.

**Table 2:** Thermodynamic parameters of non-isothermal crystallization

Sample	*Φ*/°C min^−1^	*T*_p_/°C	*T*_1/2_/min	*G*
Nylon 10T/66	5	276.39	0.71	1.41
	10	270.94	0.51	1.96
	20	263.71	0.38	2.63
	30	257.67	0.28	3.57
Nylon 10T/TiO_2_	5	278.23	0.49	2.04
	10	273.73	0.34	2.94
	20	267.62	0.25	4.00
	30	262.05	0.2	5.00
Nylon 10T/66/GF	5	277.68	0.58	1.72
	10	272.72	0.41	2.44
	20	265.63	0.30	3.33
	30	260.05	0.25	4.00
Nylon 10T/66/TiO_2_/GF	5	278.61	0.48	2.08
	10	274.37	0.33	3.03
	20	268.58	0.23	4.35
	30	264.50	0.19	5.26

#### 3.4.2 Non-isothermal crystallization kinetics

Considering the effect of TiO_2_ and GF on the crystallization behavior of nylon 10T/66, it is necessary to study the non-isothermal crystallization kinetics of nylon 10T/66/TiO_2_/GF.^14^ The relative crystallinity of nylon 10T/66/TiO_2_/GF at a certain temperature can be calculated by the ratio of the area of the crystallization curve from the initial crystallization temperature to the crystallization temperature *T* to the area of the whole crystallization peak.

**Fig. 5:**
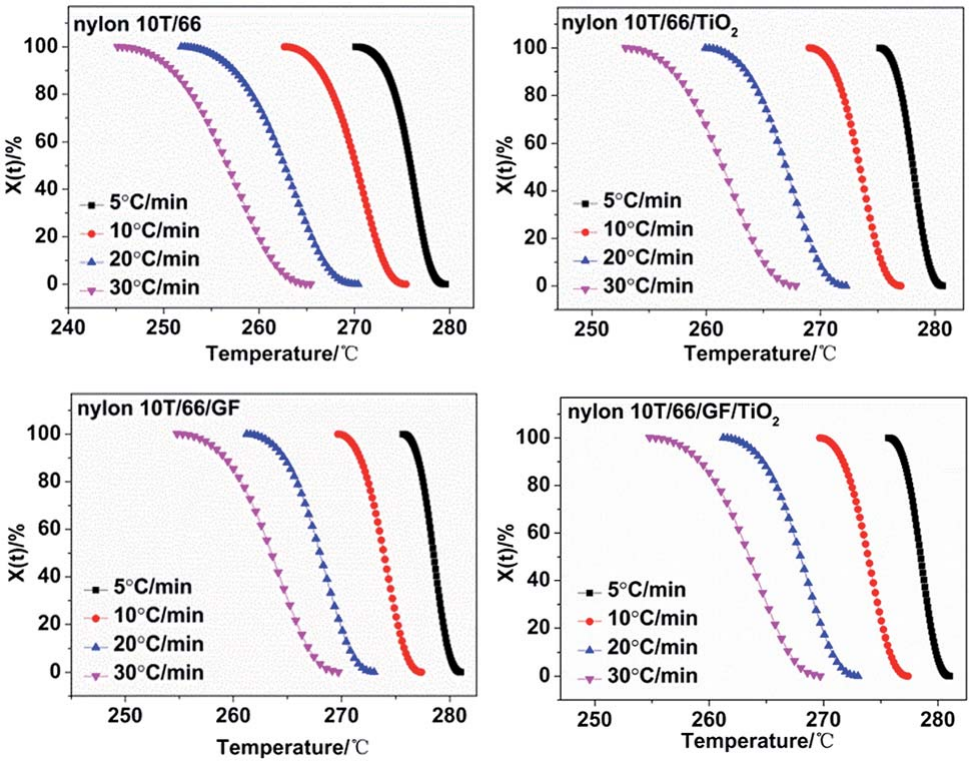
Plots of *X*_*t*_*versus T* for nylon 10T/66, nylon 10T/66/TiO_2_, nylon 10T/66/GF and nylon 10T/66/TiO_2_/GF.

Generally, the relationship between relative crystallinity and crystallization temperature can be expressed as follows:1
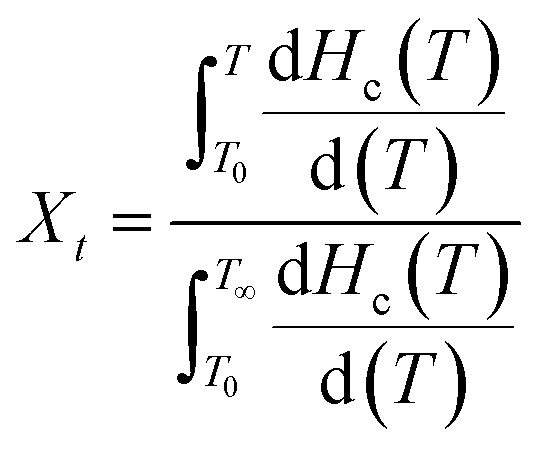
where *T*_0_ and *T*_∞_ correspond to the initial and end temperatures of the crystallization curve, respectively. And *T* represents the crystallization temperature at time *t*. Fig. 5 presents the plots of *X*_*t*_*versus T* according to eqn (1) for nylon 10T/66, nylon 10T/66/TiO_2_, nylon 10T/66/GF and nylon 10T/66/TiO_2_/GF. It can be clearly seen that all the curves show reverse S shape, implying that the cooling rate has a retardation effect on the crystallization. In addition, the lower crystallization onset temperature of all samples was obtained along with the increase of cooling rate, which could be attributed to the higher cooling rate causes the molecular chain to have insufficient time to start crystallization at a higher temperature.

**Fig. 6:**
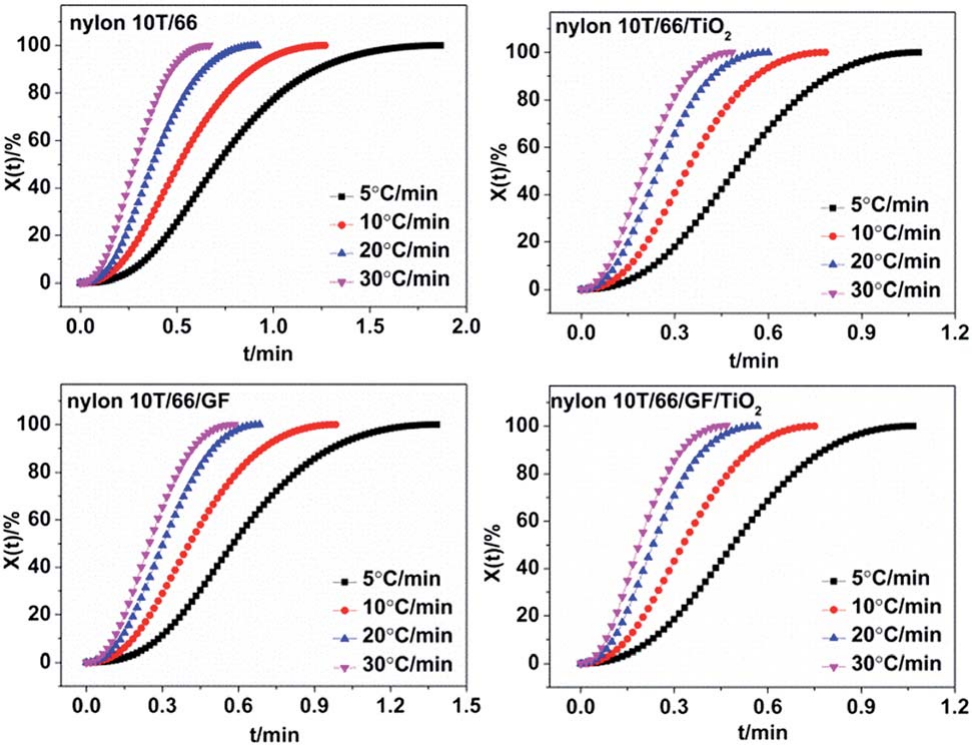
Plots of *X*_*t*_*versus t* for nylon 10T/66, nylon 10T/66/TiO_2_, nylon 10T/66/GF and nylon 10T/66/TiO_2_/GF.

The crystallization time and crystallization temperature can be transformed by the following formula:2
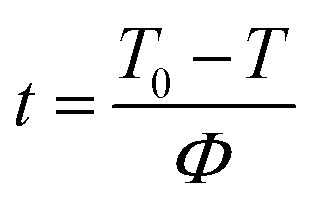
where *Φ* is the cooling rate. Combining eqn (1) and (2), we got the *X*_*t*_ = *f*(*t*) curves (Fig. 6). The time corresponding to the 50% relative crystallinity (*t*_1/2_) can be obtained from the Fig. 6. And the crystallization rate *G* = 1/*t*_1/2_, which is listed in Table 2.^21^ According to the theory of crystallization kinetics, the bigger value of *G* means the faster crystallization rate.^22^ As presented in Table 2, for each sample, *t*_1/2_ decreases and *G* increases gradually as the cooling rate increases, indicating faster crystallization rate at the high cooling rate. Interestingly, at the same cooling rate, with the addition of GF and TiO_2_, the crystallization rate increases, which further confirmed they play important roles in the hetero-geneous nucleation. Moreover, the fastest crystallization rate of nylon 10T/66/TiO_2_/GF could be ascribed to the synergistic of the TiO_2_ and GF.

As for the crystallization rate of the nylon 10T/66/TiO_2_ is faster than nylon 10T/66/GF, it is because that the smaller particle size of TiO_2_ promotes heterogeneous nucleation to a greater extent.

#### 3.4.3 Jeziorny equation

In order to study the crystallization mechanism of the materials, some equations were choosen to analysis the crystallization kinetics. Generally, Avrami equation (eqn (3)) is one of the most common methods for investigating the isothermal crystallization processes.^19^

3 *X*_*t*_ = 1 − exp(−*Z*_*t*_*t*^*n*^)

**Fig. 7:**
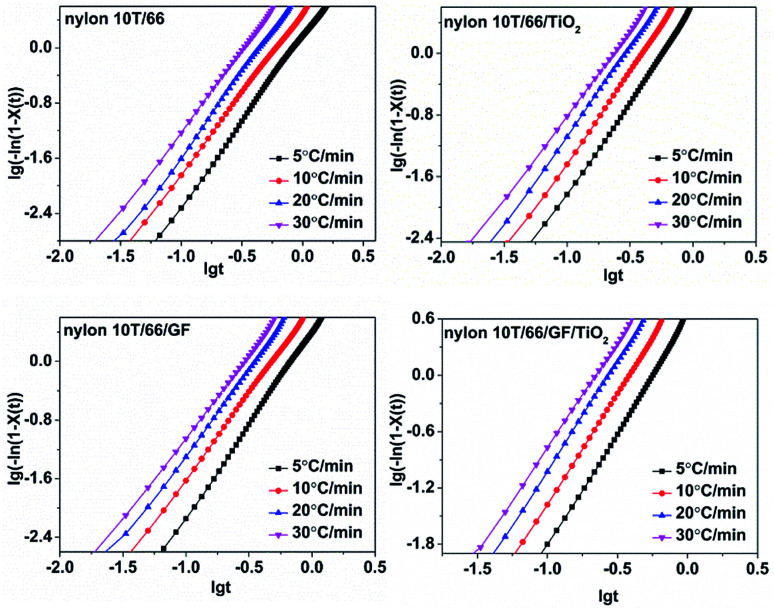
Plots of lg[−ln(1 − *X*_*t*_)] *versus* lg *t* for nylon 10T/66, nylon 10T/66/TiO_2_, nylon 10T/66/GF and nylon 10T/66/TiO_2_/GF.

Taking the logarithms of both sides, we can obtain:

4 lg[−ln(1 − *X*_*t*_)] = *n* lg *t* + lg *Z*_*t*_

where *n* is Avrami index and *Z*_*t*_ is the crystallization rate constant. Regrettably, Avrami equation is unsuited for describing non-isothermal crystallization process.^23^ In order to analysis the non-isothermal crystallization process, Jeziorny modified *Z*_*t*_ with the cooling rate, and the modified equation is as follow:^24^5
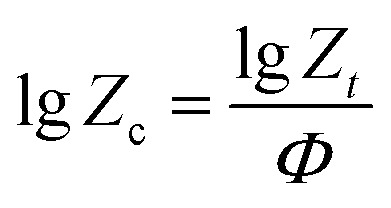
where *Z*_c_ denotes non-isothermal crystallization rate constant. On the basis of eqn (4), the plots of lg[−ln(1 − *X*_*t*_)] against lg *t* at different cooling rates are shown in Fig. 7. The *n* and *Z*_c_ can be determined from the slope and intercept, and they are presented in Table 3. As listed in Table 3, the *n* for nylon 10T/66, nylon 10T/66/TiO_2_, nylon 10T/66/GF and nylon 10T/66/TiO_2_/GF ranges from 2.17 to 2.44, 1.84 to 2.22, 2.09 to 2.29 and 1.89 to 2.29, which indicates that the mode of the nucleation and growth for these nylons may be one-dimensional and two-dimensional coexistence. As we all know, larger *Z*_c_ value corresponds to faster crystallization rate.^24^ The *Z*_c_ increased gradually when increasing the cooling rate, meaning the crystallization rate increases. For a given cooling rate, the *Z*_c_ values of nylon 10T/66/TiO_2_ and nylon 10T/66/GF are both higher than nylon 10T/66. This indicates that the TiO_2_ and GF are efficient in accelerating the crystallization nylon 10T/66. In addition, the nylon 10T/66/TiO_2_/GF has the fastest crystallization rate due to the synergistic of the TiO_2_ and GF. All these conclusions are consistent with the previous results of *G*.

**Table 3:** The parameters determined by Jeziorny method

Sample	*Φ*/°C min^−1^	*n*	*Z*_*t*_	*Z*_c_
Nylon 10T/66	5	2.27	0.90	0.98
	10	2.17	2.03	1.07
	20	2.34	5.58	1.09
	30	2.44	16.22	1.10
Nylon 10T/TiO_2_	5	2.02	1.45	1.08
	10	1.84	2.63	1.10
	20	2.14	8.51	1.11
	30	2.22	25.70	1.11
Nylon 10T/66/GF	5	2.29	1.42	1.07
	10	2.08	2.24	1.08
	20	2.09	6.31	1.10
	30	2.27	16.60	1.10
Nylon 10T/66/TiO_2_/GF	5	2.03	1.51	1.09
	10	1.89	3.16	1.12
	20	2.08	11.22	1.13
	30	2.29	33.88	1.13

#### 3.4.4 Mo equation

Ozawa equation, another method to depict crystallization, has been proved by many articles to be unsuitable for describing non-isothermal crystallization of polymers.^25,26^ However, Mo *et al.* proposed a new method (eqn (6)) combining Avrami and the Ozawa equations, which could describe the non-isothermal crystallization kinetics more accurately.^27,28^

6 lg *Z*_*t*_ + *n* lg *t* = lg *K*(*T*) − *m* lg *Φ*

**Fig. 8:**
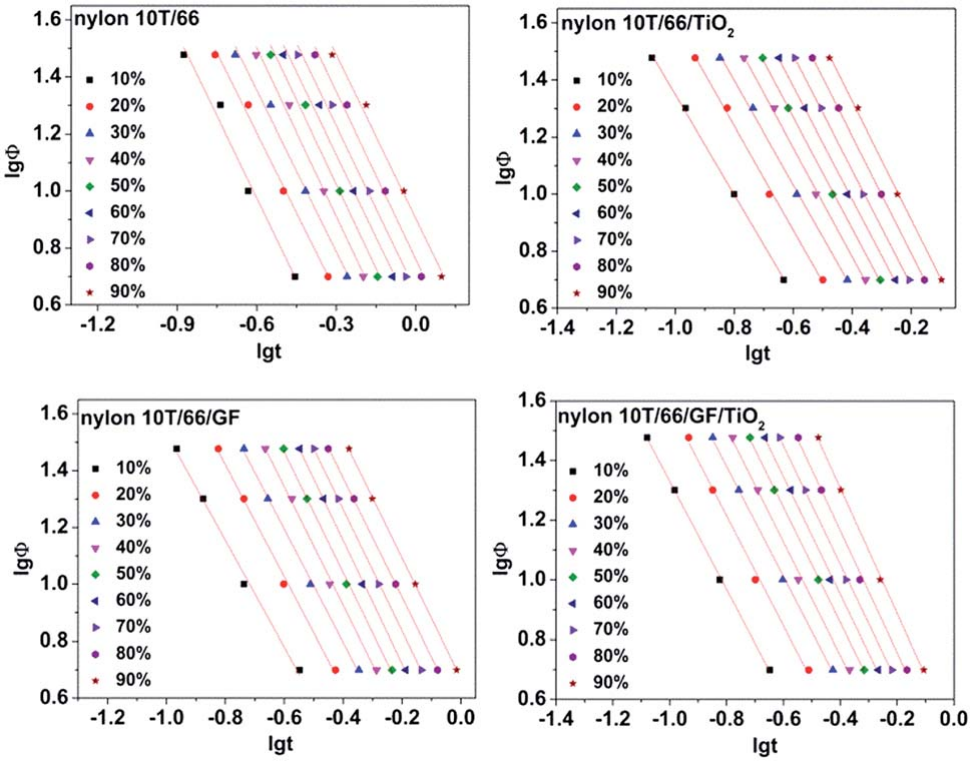
Plots of lg *Φ versus* lg *t* for nylon 10T/66, nylon 10T/66/TiO_2_, nylon 10T/66/GF and nylon 10T/66/TiO_2_/GF.

7 lg *Φ* = lg *F*(*T*) − *α* lg *t*

where *α* = *n*/*m* (*n*, Avrami exponent and *m*, Ozawa exponent) and *F*(*T*) represents the cooling rate required to reach a relative crystallinity at a unit time.^29^ Many studies have identified that Mo equation could well describe the non-isothermal crystallization behaviors of polymers, such as the poly(vinyl alcohol)/starch composite,^30^ poly(ethylene terephthalate) composites^31^ and poly(butylene succinate) (PBS).^20^Fig. 8 shows the curves of lg *Φ versus* lg *t* of nylon 10T/66, nylon 10T/66/TiO_2_, nylon 10T/66/GF and nylon 10T/66/TiO_2_/GF with a series of straight lines (the correlation coefficient *R*^2^ is shown in Table 4, they are all less than 0.99), which indicates the Mo equation is also suitable for analyzing the non-isothermal crystallization behaviors of these nylons. The *α* and *F*(*T*) could be obtained from the slopes and the intercepts, and the values are listed in Table 4. Usually, the bigger *F*(*T*) value means the slower crystallization rate.^22^ It can be clearly seen from Table 4 that the values of *F*(*T*) increases with the relative crystallinity, which indicates that if we want to reach a large relative crystallinity within a certain period of time, we must achieve this by increasing the cooling rate. For the same relative crystallinity, the *F*(*T*) values of nylon 10T/66/GF and nylon 10T/66/TiO_2_ are both lower than that of nylon 10T/66, and the value of nylon 10T/66/TiO_2_/GF is the lowest. These indicate that nylon 10T/66/GF and nylon 10T/66/TiO_2_ have faster crystallization rate than nylon 10T/66, and nylon 10T/66/TiO_2_/GF has the fastest crystallization rate, which are in perfect accordance with the results of the Jeziorny analysis.

**Table 4:** Parameters of non-isothermal crystallization kinetics by the Mo equation

Sample	*X*(*t*) (%)	*α*	*F*(*t*)	*R* ^2^
Nylon 10T/66	10	1.92	0.66	0.97
	20	1.87	1.21	0.99
	30	1.89	1.66	0.99
	40	1.97	2.09	0.99
	50	1.97	2.70	0.98
	60	1.93	3.52	0.98
	70	1.94	4.44	0.99
	80	1.96	5.75	0.99
	90	1.91	8.00	0.99
Nylon 10T/TiO_2_	10	1.75	0.39	1.00
	20	1.82	0.60	1.00
	30	1.83	0.87	1.00
	40	1.91	1.04	1.00
	50	1.95	1.25	1.00
	60	1.98	1.55	1.00
	70	2.02	1.89	1.00
	80	2.05	2.41	1.00
	90	2.07	3.14	1.00
Nylon 10T/66F	10	1.88	0.45	0.99
	20	1.97	0.70	0.99
	30	2.00	0.99	1.00
	40	2.09	1.23	1.00
	50	2.12	1.56	1.00
	60	2.17	1.93	1.00
	70	2.14	2.55	1.00
	80	2.10	3.42	1.00
	90	2.12	4.66	1.00
Nylon 10T/66/TiO_2_/GF	10	1.81	0.33	1.00
	20	1.85	0.55	0.99
	30	1.85	0.80	1.00
	40	1.91	0.96	1.00
	50	1.93	1.21	1.00
	60	1.97	1.45	1.00
	70	1.99	1.81	1.00
	80	2.04	2.23	1.00
	90	2.10	2.94	1.00

#### 3.4.5 Crystallization activation energy

Activation energy is an important parameter in the phase transition process, which is related to the energy and barrier of phase transition.^32^ Activation energy could effectively reflect the crystallization ability of polymers.^33^

**Fig. 9:**
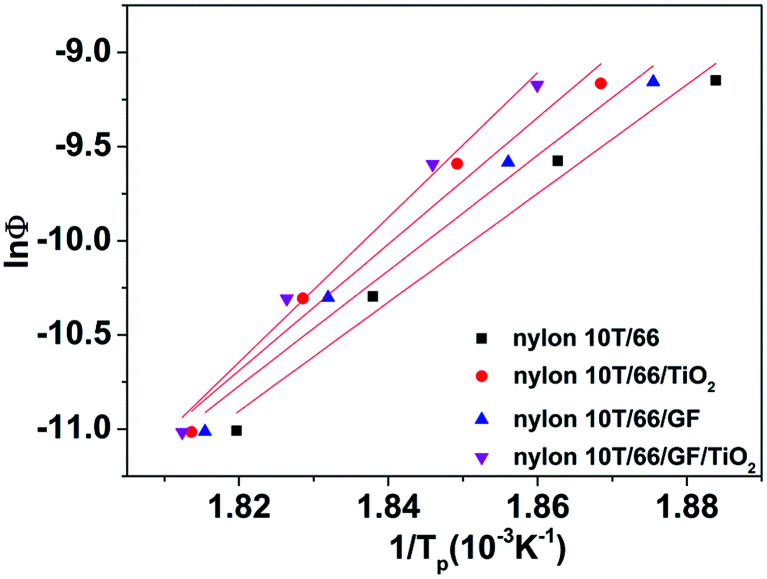
Plots of ln(*Φ*/*T*_p_^2^) *versus* 1/*T*_p_.

Kissinger^34^ is the most common method for calculating crystallization activation energy.^35^8
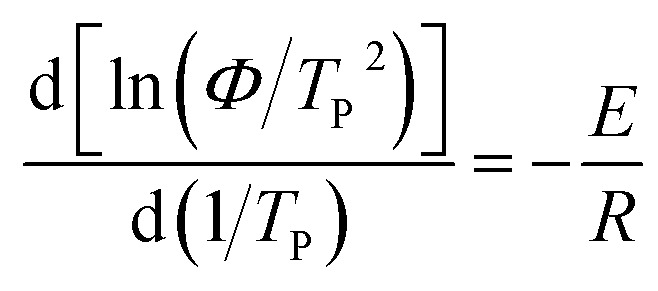
where *E* denotes the activation energy. The plots of ln(*Φ*/*T*_p_^2^) *versus* 1/*T*_p_ is shown in Fig. 9. The activation energy could be calculated from the slope of the plots (see Table 5). As listed in Table 5, the *R*^2^ of these samples are less than 0.97, which mean that the lines have a good linear relationship. It also can be clearly found that adding GF and TiO_2_ reduces the *E* value and the *E* value of nylon 10T/66/TiO_2_/GF is the lowest. In other word, adding GF and TiO_2_ improves the crystallization rate of nylon 10T/66 and nylon 10T/66/TiO_2_/GF has the most fast crystallization rate.

**Table 5:** Activation energy determined by Kissinger

Sample	Nylon 10T/66	Nylon 10T/TiO_2_	Nylon 10T/66 /GF	Nylon 10T/66/TiO_2_/GF
*E*/(kJ mol^−1^)	−240.23	−279.38	−254.82	−319.67
*R* ^2^	0.97	0.97	0.97	0.98

### 3.5 XRD analysis

**Fig. 10:**
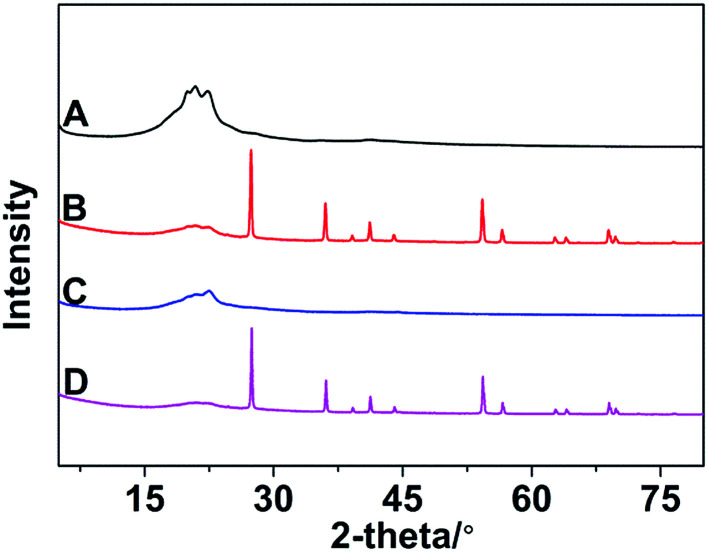
The XRD patterns of nylon 10T66 (A), nylon 10T/66/TiO_2_ (B), nylon 10T/66/GF (C) and nylon 10T/66/TiO_2_/GF (D).

The XRD patterns of different samples are showed in Fig. 10. It can be clearly seen that broad diffraction peaks which belong to crystal of nylon 10T/66 appeared at about 20° in all samples. Other poignant peaks are compounding to the rutile TiO_2_ existing in nylon 10T/66/TiO_2_ and nylon 10T/66/TiO_2_/GF. And there are no diffraction peaks caused by GF both in nylon 10T/66/GF and nylon 10T/66/TiO_2_/GF owing the cladding of outer substrates.

### 3.6 Color comparison and yellowing resistance

**Fig. 11:**
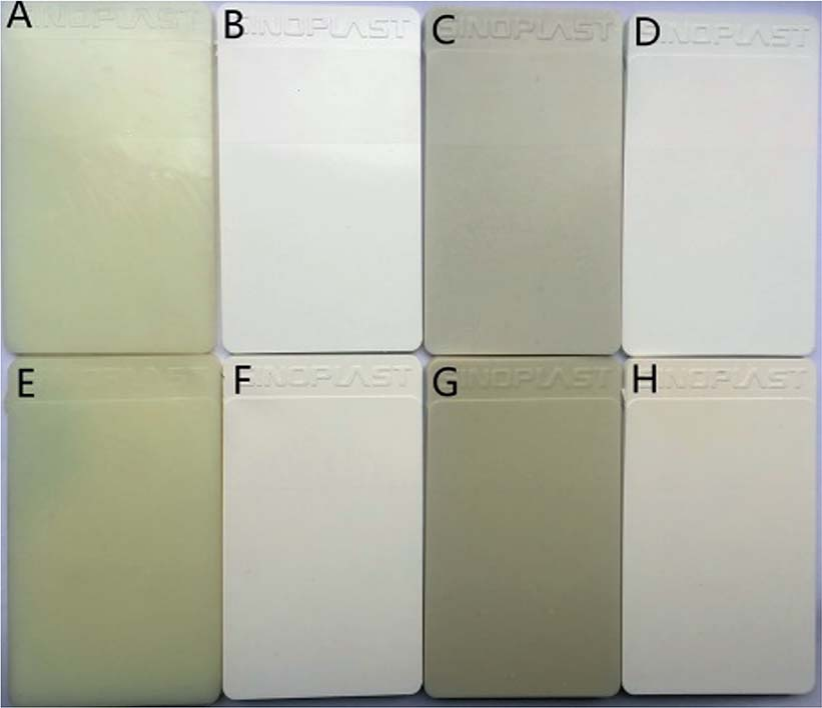
Plates of nylon 10T/66 (A and E), nylon 10T/66/TiO_2_ (B and F), nylon 10T/66/GF (C and G) and nylon 10T/66/TiO_2_/GF (D and H) (0 h: A–D, 2 h: E–H).

Fig. 11 is the color plates the nylon 10T/66, nylon 10T/66/TiO_2_, nylon 10T/66/GF and nylon 10T/66/TiO_2_/GF after 0 or 2 hours of thermal oxidation aging at 180 °C. It can be clearly seen from Fig. 11 that the addition of TiO_2_ can significantly improve the whiteness of nylon 10T/66 whether 0 or 2 hours of thermal oxygen aging. Although the whiteness of nylon 10T/66/TiO_2_/GF has a small decrease compared with nylon 10T/66/TiO_2_, it still has a significant improvement over nylon 10T/66.

Table 6 shows the comparison of *L* and *b* values for different samples. The larger the *L* and the *b* values, the whiter and yellower color of the material, respectively.^36,37^ As shown in Table 6, nylon 10T/66/TiO_2_/GF has lager *L* values and smaller *b* values than nylon 10T/66 whether it is 0 or 2 hours of the hot oxygen aging, which means that the color of the nylon 10T/66/TiO_2_/GF is whiter. This result is same as the visual observation of Fig. 11.

**Table 6:** The comparison of *L* and *b* values for different samples

Sample		Nylon 10T/66	Nylon 10T/66/TiO_2_	Nylon 10T/66/GF	Nylon 10T/66/TiO_2_/GF
*L*	0 h	82.20	97.16	79.37	95.04
	2 h	80.53	96.01	77.91	93.76
*b*	0 h	10.24	2.93	9.43	2.99
	2 h	13.34	5.49	13.20	5.75
	Δ*b*	3.1	2.56	3.77	2.76

In addition, the degree of yellowing, an important indicator of the heat-resistant plastics, could be well reflected by the magnitude of the change in Δ*b* value, which is equal to *b* (2 h) minus *b* (0 h). As shown in Table 6, the Δ*b* value of nylon 10T/66/TiO_2_/GF is significantly lower than that of neat nylon 10T/66, indicating that nylon 10T/66/TiO_2_/GF has better yellowing resistance neat nylon 10T/66.

### 3.7 Mechanical properties

The mechanical performance parameters of the nylon 10T/66, nylon 10T/66/TiO_2_, nylon 10T/66/GF and nylon 10T/66/TiO_2_/GF are summarized in Table 7. It can be observed that the tensile strength, elongation at break and impact strength of nylon 10T/66 decreased with the addition of TiO_2_. However, the tensile strength, flexural strength and flexural modulus are obviously improved by introducing GF, while the elongation at break and impact strength are comparable to nylon 10T/66. These phenomena are attributed that the addition of GF promotes the crystallization and increases surface fracture energy of nylon. Also, it is due to the rigidization effect of GF within the nylon and higher modulus of GF as compared with nylon.^8,9^ Clearly, although the mechanical properties of nylon 10T/66/TiO_2_/GF are lower than those of nylon 10T/66/GF, there is still a significant increase compared with neat nylon 10T/66.

**Table 7:** The mechanical properties of different samples

Samples	Tensile strength [MPa]	Breaking elongation [%]	Bending strength [MPa]	Bending modulus [MPa]	Impact strength [kJ m^2^]
Nylon 10T/66	49.56 ± 3.54	3.66 ± 0.33	89.79 ± 2.9	2375 ± 63.73	5.9 ± 0.4
Nylon 10T/66/TiO_2_	43.26 ± 2.49	3.06 ± 0.15	93.14 ± 4.45	2880 ± 77.78	4.7 ± 0.14
Nylon 10T/66/GF	98.96 ± 1.73	3.99 ± 0.1	141.79 ± 2.12	5845 ± 0	5.51 ± 0.35
Nylon 10T/66/TiO_2_/GF	72.59 ± 5.65	3.19 ± 0.15	129.29 ± 1.41	5640 ± 91.92	4.5 ± 0.49

## 4. Conclusions

In this paper, we prepared nylon 10T/66/TiO_2_/GF by blending as-synthesised nylon 10T/66 with TiO_2_ and GF. The non-isothermal crystallization behaviors of nylon 10T/66, nylon 10T/66/TiO_2_, nylon 10T/66/GF and nylon 10T/66/TiO_2_/GF were investigated. The crystallization curves showed that nylon 10T/66/TiO_2_/GF has a higher crystallization temperature than nylon 10T/66. The crystallization rate *G*, Jeziorny and Mo analysis revealed that nylon 10T/66/TiO_2_/GF has higher crystallization rate. The Kissinger method was used to calculate the non-isothermal crystallization activation energies, indicating that the *E* value of nylon 10T/66/TiO_2_/GF is lower than nylon 10T/66. All these results can be attributed to that GF and TiO_2_ play strong crucial roles in the heterogeneous nucleation. The color comparison and mechanical properties showed that the yellowing resistance and mechanical properties of nylon 10T/66/TiO_2_/GF were better than nylon 10T/66. The nylon 10T/66/TiO_2_/GF composites possesses higher crystallization temperature, crystallization rate, whiter color, better yellowing resistance and mechanical properties, and has promising applicability in the field of LED lights.

## Conflicts of interest

There are no conflicts to declare.

## Supplementary Material
